# Horseradish Essential Oil as a Promising Anti-Algal Product for Prevention of Phytoplankton Proliferation and Biofouling

**DOI:** 10.3390/plants10081550

**Published:** 2021-07-28

**Authors:** István Bácsi, Sándor Gonda, Zsuzsanna Nemes-Kókai, Viktória B-Béres, Gábor Vasas

**Affiliations:** 1 Department of Hydrobiology, University of Debrecen, Egyetem tér 1, H-4032 Debrecen, Hungary; 2 Department of Botany, University of Debrecen, Egyetem tér 1, H-4032 Debrecen, Hungary; gondasandor@gmail.com (S.G.); vasas.gabor@science.unideb.hu (G.V.); 3 Centre for Ecological Research, Institute of Aquatic Ecology, Department of Tisza Research, Bem tér 1, H-4026 Debrecen, Hungary; kokai.zsuzsanna@ecolres.hu (Z.N.-K.); beres.viktoria@gmail.com (V.B.-B.); 4 Department of Ecology, University of Debrecen, Egyetem tér 1, H-4032 Debrecen, Hungary; 5 Pál Juhász-Nagy Doctoral School of Biology and Environmental Sciences, University of Debrecen, Egyetem tér 1, H-4032 Debrecen, Hungary

**Keywords:** horseradish essential oil, cyanobacteria, eukaryotic algae, phytoplankton, biofilm

## Abstract

Increased proliferation of algae is a current problem in natural and artificial water bodies. Controlling nutrients is the most sustainable treatment of increased algal proliferation, however in certain cases, it is not sufficiently available, or it does not provide results fast enough. Chemicals derived from natural sources, which could be effective in low concentrations and are biodegradable, may have an advantage over conventional chemical treatments. The main aim of the present study was to investigate the anti-cyanobacterial and anti-algal properties of allyl-isothiocyanate-containing essential oil produced from horseradish roots with a complex approach of the topic: on laboratory strains of cyanobacteria and eukaryotic algae, on microcosms containing natural phytoplankton assemblages, and on semi-natural biofilms. The results show that acute treatment can significantly reduce the viability of all the tested cyanobacteria and eukaryotic algae. Results of microcosm experiments with natural phytoplankton assemblages show that horseradish essential oil from 7.1 × 10^−6^% (*v/v*) is applicable to push back phytoplankton proliferation even in natural assemblages. The individual number in the biofilm was dropped down to one-fifth of the original individual number, so 7.1 × 10^−6^% (*v/v*) and higher concentration of the essential oil can be considered as a successful treatment against biofouling.

## 1. Introduction

Increased proliferation of algae is a current problem in natural as well as in established water bodies (beaches, fishponds, urban ponds, pools, fountains, etc.). The most obvious consequence of the mass development of algae is that they cause difficulties in water usage in many areas (recreation, fisheries, agriculture, industry, and drinking water [1]). Phytoplankton blooms often come along with the production of unpleasant odor, which could be problematic in urban environments, but more importantly, blooms can cause oxygen depletion in water bodies, they may be toxic (due to the production of biochemically active secondary metabolites); consequently, blooms can cause perish of other organisms, leading to changes in diversity [2].

In addition to planktic algae, benthic algae can also appear with high biomass in ornamental ponds, pools, fountains, wells as part of biofouling (microfouling [3]). Biofouling is a strongly disadvantageous phenomenon that may cause several problems on artificial surfaces immersed in water [4]. Algae may appear even in land conditions (walls, fences, roads) with the presence of adequate humidity, causing aesthetic, moreover economic, or even health problems.

The decontamination of artificial pools and fountains seems to be a simpler task, as in these cases, there are no living organisms to be taken into account. However, human health impacts and the environmental impacts of effluents after chemical treatment should also be considered in these cases. The situation is more difficult in the case of semi-natural or natural water bodies (beaches, fishponds, urban ponds), where the presence of living communities must also be taken into account when developing measures designed to reduce the growth of algae.

There are many chemical methods (compounds) that are used to reduce the proliferation of phytoplankton in water bodies either directly via growth inhibition or indirectly by a decrease in nutrient concentration [2]. Similarly, paints and coatings containing toxic materials are commonly used to control biofouling [4].

Metal (aluminum, iron, or copper)-containing compounds are used either as direct algaecides or as the most common coagulants for removal of nutrients (mainly phosphorous) from waters in many areas of the water industry [5,6,7,8,9,10,11,12]. Besides copper, antifouling paints or coatings usually contained lead, arsenic, or mercury [13]. Calcium-containing and clay compounds [14,15,16,17] are also usually used as inorganic coagulants. The best-known metal-containing antifoulants are organotin compounds [18]. The main problem of the application of metal-containing compounds is the possible toxic effect on non-target organisms and the resulting, also possibly toxic sludge [2,19].

Other possible methods using chemicals are able to generate different reactive oxygen species (ROS) upon light irradiation. Hydrogen peroxide, phtalocyanines [20,21], titanium-dioxide [2], chlorine [22], hypochlorite or chlorine-dioxide [23], permanganate [24,25,26] or ozone [27] are the most known compounds used for generating ROS. Cyanobacteria seem to be significantly more sensitive to the presence of ROS than other aquatic organisms [20,21], which could be advantageous in controlling cyanobacterial blooms. There are some disadvantages of these compounds too: toxic effects to non-target organisms, insufficient solubility [2], formation of undesirable by-products, or unpleasant odor occurrences [17].

The third group of chemicals used in algal proliferation control is the group of organic herbicides. Diuron (3-(3,4-dichlorophenyl)-1,1-dimethylurea) [28] and Endothall (3,6-endoxohexahydrophthalic acid) are well known and relatively widely used herbicides in aquatic habitats [29]. Unfortunately, the use of neither compound is problem-free [30,31,32].

Chemical compounds derived or extracted from living organisms seem to be a promising alternative among methods against undesirable algal proliferation [2]. Over the last few decades, many compounds have been isolated from microalgae, macroalgae, and aquatic microorganisms [33,34,35] or from aquatic (marine) invertebrates [3]. Recently, plant metabolites as biocides came to the fore [36] since plant secondary metabolites, such as essential oils and herb extracts, have revealed relatively powerful broad-spectrum antimicrobial activities [37,38,39]. One of the plants as a promising source of antimicrobial metabolites is *Armoracia rusticana*, commonly known as horseradish.

Horseradish has been cultivated for a long time and is used primarily in the food industry, but more and more research data highlight other possible uses of this plant [40]. Glucosinolates belong to the most important secondary metabolites of horseradish [41]. These compounds are part of the plant’s defense mechanisms: when damage is done to the plant, hydrolysis of glucosinolates by the enzyme myrosinase is triggered. The quality of hydrolysis products depends on many factors. Isothiocyanates (ITCs) are the default main products [42,43,44]. ITCs are promising antimicrobial agents: their antibacterial, antiprotozoal [45,46,47,48], and antifungal [49] effects have been reported. Enzyme inactivation [46], uncoupling action of oxidative phosphorylation [45], oxidative stress, and DNA damage [50] are in the background of antimicrobial activity of isothiocyanates.

The main aim of the present study was to investigate the anti-algal properties of allyl-isothiocynate (AITC)- and phenetyl-isothiocyanate (PEITC)-containing essential oil produced from horseradish roots. The goal was a complex approach to the topic: model organisms representing potential water bloom-forming species were applied in laboratory experiments, as well as natural phytoplankton assemblages in microcosms and biofouling on artificial surfaces also were studied. This complex approach provides the opportunity to evaluate the possible differences in model systems and in actual assemblages and to study the anti-algal, anti-bloom-forming, and antifouling effects of the horseradish essential oil. We aimed to answer the following study questions:

How sensitive are laboratory strains of common, potentially bloom-forming cyanobacteria and eukaryotic algae to horseradish essential oil? What are the concentrations and exposition times causing 50% growth inhibition?

How does horseradish essential oil extract work in a more life-like environment? What are the effects on natural algal assemblages in microcosms?

How effective is the horseradish essential oil extract treatment on semi-natural benthic algal communities (algal biofilms, antifouling effect)?

## 2. Results

### 2.1. Laboratory Experiments

**Figure 1:**
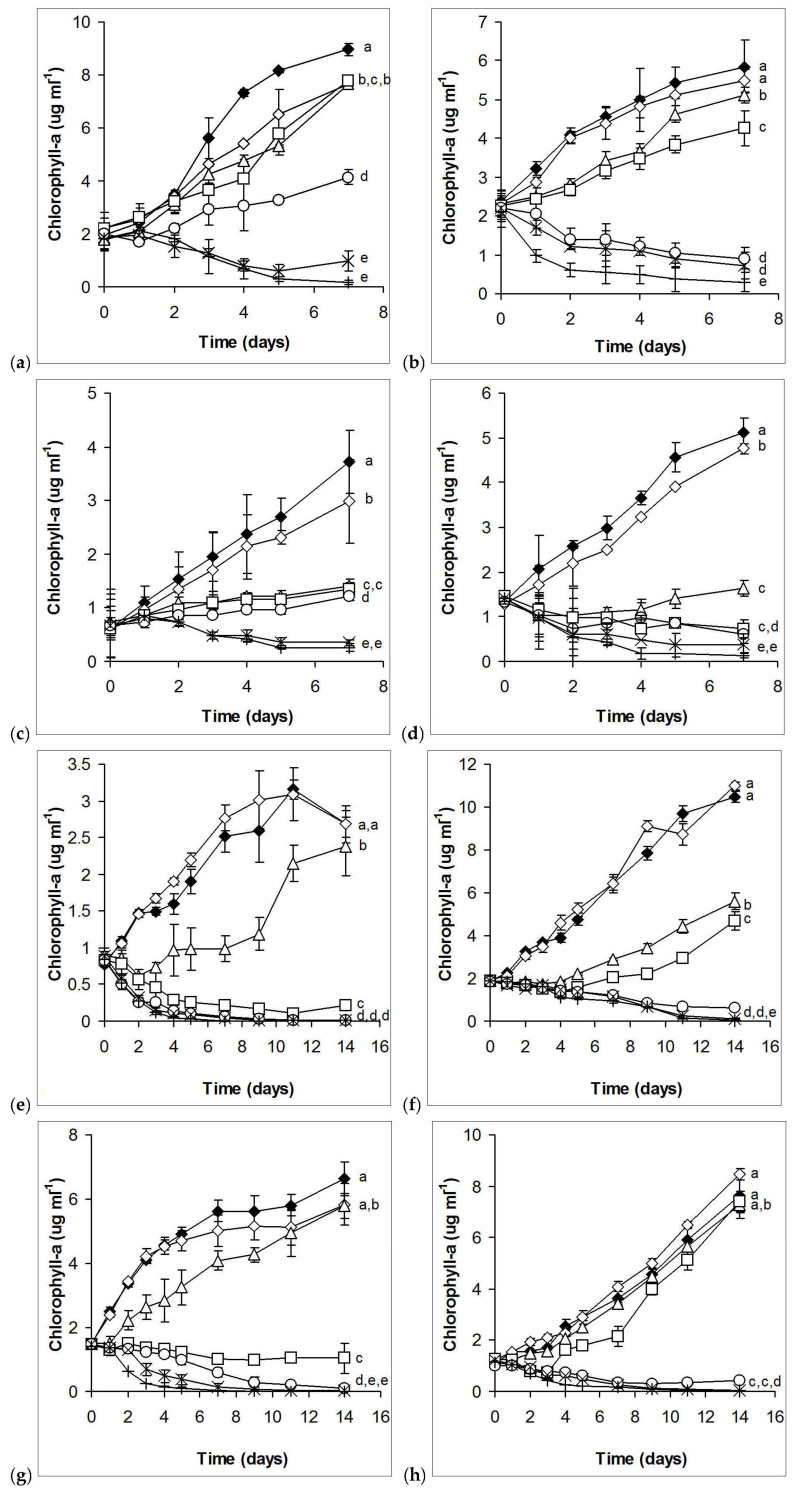
Growth of cyanobacteria and eukaryotic algae in control and horseradish essential oil-treated cultures. (**a**): *Synechococcus elongatus*; (**b**): *Microcystis aeruginosa*; (**c**): *Cylindrospermopsis raciborskii*; (**d**): *Dunaliella salina*; (**e**): *Cryptomonas ovata*; (**f**): *Chlorella sorokiniana*; (**g**): *Chlorococcum* sp.; (**h**): *Desmodesmus communis*. Black diamonds: control; empty diamonds: 28.6 × 10^−6^ % (v/v) ethanol; empty triangles: 1.8 × 10^−6^; empty squares: 3.6 × 10^−6^; empty circles: 7.1 × 10^−6^; exes: 14.3 × 10^−6^; plus signs: 28.6 × 10^−6^ % (v/v) horseradish essential oil. Mean values ±standard deviations are plotted (n = 3). Treatments not sharing the same lowercase letters are significantly different (*p* < 0.05).

Considering growth, 1.8 – 3.6 × 10^−6^% essential oil caused stronger growth inhibition of the filamentous cyanobacterium *Cylindrospermopsis raciborskii* cultures than to the unicellular ones (*Synechococcus elongatus* and *Microcystis aeruginosa*) after 7 days of exposition. However, higher concentrations (14.3 – 28.6 × 10^−6^%) caused weaker growth inhibition in cultures of the filamentous species than in cultures of the unicellular ones (Figure 1a–c). On the basis of EC_50_ values (both for the fourth and seventh days), *Synechococcus* showed higher tolerance against horseradish essential oil than the other two cyanobacterial species (Table 1). There were no significant differences among EC_50_ values calculated for the fourth and seventh days of either cyanobacterial species.

The flagellated, salt-tolerant (or halophillic) green alga *Dunaliella salina* showed the highest sensitivity among eukaryotic algae (Figure 1d). Stronger growth inhibition occurred on the fourth and seventh day for the cell wall-less *Cryptomonas ovata* than for the coccoid green alga *Chlorella sorokiniana* (Figure 1e,f), but more or less similar EC_50_ values were calculated (Table 2). The unicellular *Chlorococcum* sp. and the coenobial *Desmodesmus communis* were the most tolerant ones among eukaryotic algae, only 7.1 × 10^−6^% or more horseradish essential oil caused permanent growth inhibition, without observable growth regeneration (Figure 1g,h). Increasing EC_50_ values from day 7 to day 14 indicates increasing tolerance in the second week of exposure in the case of the cryptomonad and the different green algae (except *D. salina*; Table 1).

Although the lower sensitivity of cyanobacteria is not entirely obvious on the basis of the extent of growth inhibitions on certain days, EC_50_ values clearly indicate that cyanobacteria have higher tolerance against horseradish essential oil than eukaryotic algae. On the other hand, it has to be emphasized that growth regenerations also occurred in the case of green algae from the fourth day or later in cultures treated with lower concentrations (1.8 – 3.6 × 10^−6^%), as was already mentioned above.

### 2.2. Phytoplankton Assemblages in Microcosm Experiments

Physical-chemical parameters changed similarly in control and in essential oil-treated assemblages (data not shown). Volume losses because of evaporation were below 1% in all microcosms throughout the experiment. Relative abundances of zooplankton species (mainly rotifers) and protozoan grazers (mainly ciliates) did not reach 2% of all the observed specimens during the experiments.

**Figure 2:**
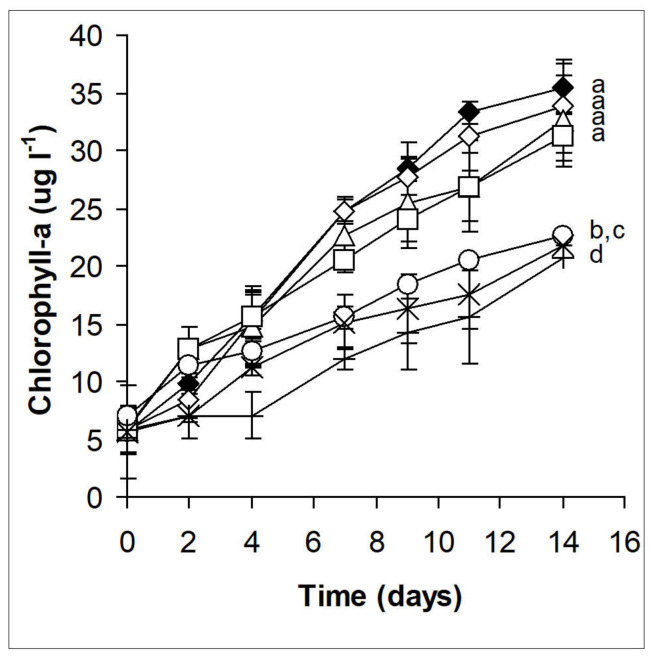
Quantitative changes of phytoplankton in microcosm experiments based on chlorophyll-a content as a result of horseradish essential oil treatment. Black diamonds: control; empty diamonds: 28.6 × 10^−6^ % (v/v) ethanol; empty triangles: 1.8 × 10^−6^; empty squares: 3.6 × 10^−6^; empty circles: 7.1 × 10^−6^; exes: 14.3 × 10^−6^; plus signs: 28.6 × 10^−6^ % (v/v) horseradish essential oil. Mean values ±standard deviations are plotted (n = 3). Treatments not sharing the same lowercase letters are significantly different (*p* < 0.05).

Quantitative changes in phytoplankton assemblages were assessed on the basis of chlorophyll-a content changes. Ethanol used to dilute the essential oil alone did not significantly affect the chlorophyll content, nor did essential oil concentrations in the range of 1.8 – 3.6 × 10^−6^ % (Figure 2). Higher essential oil concentrations (ranging 7.1 – 28.6 × 10^−6^ %) resulted in significantly lower chlorophyll-a content compared to the control and from other treatments (*p* <0.01; Figure 2). However, it should be noted that chlorophyll-a content increased even in the presence of the highest essential oil concentration (Figure 2). EC_50_ values were 26.67 ± 2.51 × 10^−6^ % for the 4^th^ day; 25.43 ± 4.46 × 10^−6^ % for the 7^th^ day, and 83.83 ± 32.21 × 10^−6^ % for the 14^th^ day. Significant (*p* <0.05) increase in EC_50_ to the 14^th^ day indicates decreasing the sensitivity of the assemblages, regeneration of phytoplankton growth.

**Figure 3:**
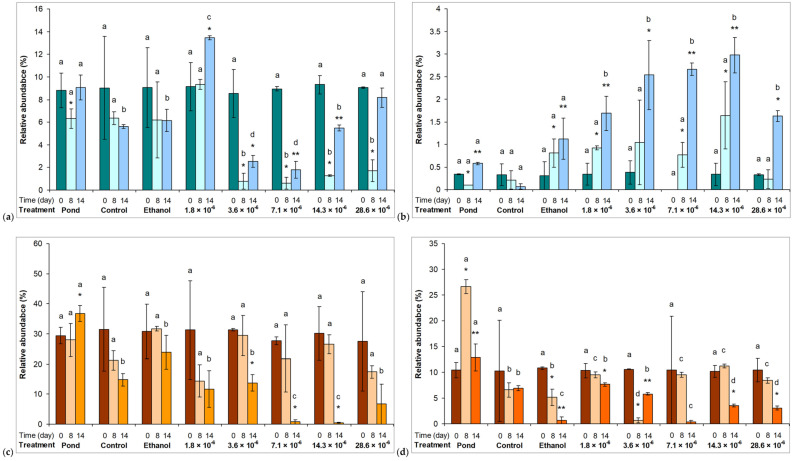
Relative abundances of the main cyanobacterium ((**a**,**b**); Cyano 1 - Synechococcales and 2 - Chroococcales) and diatom (**c**,**d**); Diatom 1 - Bacillariophyceae and 2 - Mediophyceae) groups in the pond, in control and in treated microcosm assemblages. Control: untreated microcosm. Ethanol: the microcosm contained 28.6 × 10^−6^ % (v/v) ethanol. 0, 8, and 14 are the sampling days; 1.8 – 28.6 × 10^−6^ are the applied horseradish essential oil concentrations (%, v/v; dissolved in ethanol). Mean values ±standard deviations are plotted (n = 3). Significant differences on a given day among treatments are indicated with different lowercase letters. Significant differences among days within a treatment are indicated with asterisks.

Compositional changes were assessed on the basis of relative abundance changes of the main taxonomical groups. The most commonly observed genera of all individual taxa are shown in Table S1. The most striking change was the significant decrease in the relative abundance of Cyano 1 group to the seventh day of the exposure in assemblages treated with 3.6 × 10^−6^ % or higher essential oil concentrations (*p* < 0.01; Figure 3a). Although some extent of recovery to the 14^th^ day could be observed, the relative abundance of the Cyano 1 group remained significantly lower (*p* < 0.01) in certain essential oil treatments than in other assemblages (Figure 3a). The other group of cyanobacteria represented by low relative abundance (Cyano 2 group) was not sensitive to the presence of the horseradish essential oil (Figure 3b). Significant decreases in the relative abundances of diatoms (Diatom 1 and 2 groups) to the 14^th^ day are also worth mentioning (*p* < 0.05; Figure 3c,d).

**Figure 4:**
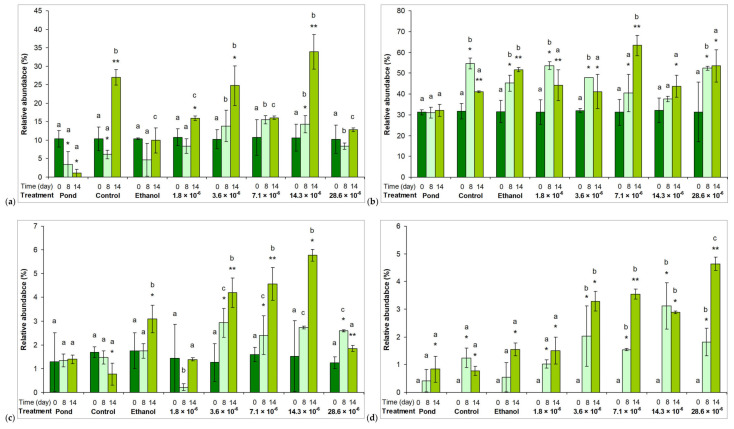
Relative abundances of the main green algae groups ((**a**–**d**); Green 1 - Trebouxiophyceae, 2 - Chlorophyceae, 3 - Conjugatophyceae, and 4 - Carophyceae + Klebsormidiophyceae) in the pond, in control and in treated microcosm assemblages. Control: untreated microcosm. Ethanol: the microcosm contained 28.6 × 10^−6^ % (v/v) ethanol. 0, 8, and 14 are the sampling days; 1.8 – 28.6 × 10^−6^ are the applied horseradish essential oil concentrations (%, v/v; dissolved in ethanol). Mean values ±standard deviations are plotted (n = 3). Significant differences on a given day among treatments are indicated with different lowercase letters. Significant differences among days within a treatment are indicated with asterisks.

The dominant green algae were not sensitive to the presence of the horseradish essential oil. Their abundances showed increasing tendencies regardless of the applied concentrations. In fact, their relative abundances were higher in essential oil-treated assemblages (Figure 4a–d).

### 2.3. Benthic Assemblages on Artificial Surfaces

Development of the natural benthic assemblages was unhindered, and green algae appeared in greater quantities alongside brown diatoms to the 5^th^–6^th^ week of the experiment (Figure S1a). The lowest concentration of horseradish essential oil (1.8 × 10^−6^ %) did not prevent the development of the biofilm (Figure S1b); however, no significant amount of green algae seemed to appear. Higher concentrations (7.1 × 10^−6^ and 28.6 × 10^−6^ %) inhibited the formation of the biofilm, although not completely prevented it. Based on the color of the biofilms, it was assumed that green algae and cyanobacteria may have been present in larger amounts at treatments 7.1 × 10^−6^ and 28.6 × 10^−6^ % (Figure S1c,d).

**Figure 5:**
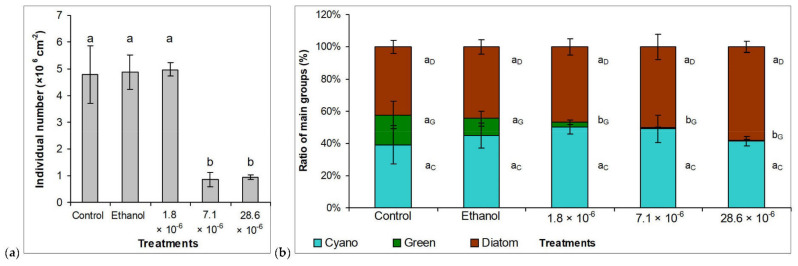
(**a**) The individual number per unit area and (**b**) proportion of the major groups (cyanobacteria, diatoms, green algae) in control and differently treated biofilms on the 6^th^ week of the experiment. Control: untreated biofilm. Ethanol: the culturing medium contained 28.6 × 10^−6^ % (v/v) ethanol. 1.8, 7.1, and 28.6 × 10^−6^: the applied essential oil concentrations (%, v/v; dissolved in ethanol). Mean values and standard deviations are indicated (n = 3). Lowercase letters indicate significant differences.

Microscopic examinations supported the “macroscopic” observations of biofilm development. Quantitative data also showed that the lowest used concentration of horseradish essential oil did not affect biofilm development, the number of individuals per unit surface area (1 cm^2^) did not show a significant difference in the 1.8 × 10^−6^ % essential oil-treated culture compared to the control (Figure 5a). However, the next used concentration (7.1 × 10^−6^ %) caused about 82% inhibition of biofilm development. Further increase in horseradish essential oil concentration (28.6 × 10^−6^ %) did not cause significant difference compared to 7.1 × 10^−6^ % treatment (80.5% inhibition; Figure 5a). EC_50_ on the 6^th^ week was 5.2 ± 0.9 × 10^−6^ v/v % horseradish essential oil.

The data on the composition of the biofilms only partially supported our assumptions based on their color change. In control biofilms, according to our assumption, the appearance of the green color was indeed the result of an increase in the proportion of green algae: their estimated proportion in the inoculum of around 10% increased to almost 19% under control conditions (Figure 5b). The species composition of the green algae did not change significantly during the 6 weeks: *Tetradesmus obliquus* was the dominant green alga species also in the mature biofilm, as it was in the original biofilm used for inoculation. The proportion of green algae decreased significantly (*p* <0.05; from 18.7% to 2.8%; Figure 5) at the lowest applied horseradish essential oil concentration (1.8× 10^−6^%). The niches freed during the fall of green algae were filled by cyanobacteria and diatoms. The proportion of both groups increased, although to a greater extent, by cyanobacteria. However, the brown pigments of diatoms (mainly fucoxanthin) suppressed the bluish-green color of cyanobacteria, which can also be seen on the images of the biofilms (Figure S1). Coccoid unicellular species among cyanobacteria and *Achnanthidium exiguum* among diatoms were present in outstanding amounts. The 7.1 × 10^−6^% essential oil concentration caused a further but non-significant decrease in the proportion of green algae (Figure 5b), i.e., the green color appearing in the biofilm cannot be related to green algae, as it was assumed. It could be observed in the 1.8 × 10^−6^% treatment that the large presence of cyanobacteria can also be masked by the brown color of “healthy” diatoms present in a similar proportion. Based on microscopic observations and quantitative data, the green color observed during the 7.1 × 10^−6^% treatment is partly due to the presence of cyanobacteria, but more to the fall of diatoms and the degradation of their brown pigments. A further increase in the essential oil concentration caused an additional but not significant decrease in the proportion of green algae and even cyanobacteria (Figure 5b). The proportion of diatoms was higher compared to the previous treatments, although the difference was not significant. The pale green color of the biofilm at the end of the exposure is thus partly due to the presence of cyanobacteria and partly due to the diatoms that have lost their brown pigments.

## 3. Discussion

Although antimicrobial activities of GLS and their hydrolysis products, especially ITCs, are quite well known [49,51], we did not find relevant literature data about the algaecide effects of these compounds. As our results show, acute treatment can significantly reduce the viability of all the tested cyanobacteria and eukaryotic algae. Cyanobacteria seemed to be less sensitive to the treatments both on the basis of chlorophyll contents and EC_50_ values. *Synechococcus* was less sensitive than the other studied cyanobacteria. Exact explanations of these phenomena require further investigations. What is currently can be concluded from the literature data is that some bacteria have a higher sensitivity to different ITCs than others [51], and their sensitivity seems not to be related to their morphology, cell wall structure, or physiological activity. In the case of the tested *Synechococcus elongatus* strain, one reason for lower sensitivity could be the fast proliferation of the species. The higher sensitivity of eukaryotes was also observed among non-photosynthetic microbes: AITC and PEITC severely inhibited certain yeasts and filamentous fungi. At the same time, the same concentration was not effective on certain Gram-positive and Gram-negative bacteria [52]. The mechanism behind the antimicrobial activities of ITCs is far from clear. It was not even precisely outlined whether ITCs exert their effects inside the prokaryotic and eukaryotic cells or their effect on cell membranes. It was observed that AITC is able to create pores on the bacterial cell membrane inducing membrane leakage [53], and inside the cells, AITC is able to react with many substances [54]. Intracellular accumulation of reactive oxygen species (ROS) and mitochondrial membrane depolarization as main effects were reported in the case of fungal cells treated with ITCs (AITC, PEITC, and benzyl-ITC - BITC; [55]). The most likely targets of ITCs are molecules with thiol groups, many of them responsible for redox homeostasis of the cells, which means that oxidative stress seems to be the main background of antimicrobial (antifungal) activity [49]. The higher sensitivity of cyanobacteria to the accumulation of ROS, especially hydrogen peroxide compared to eukaryotic algae, is known [56], although our laboratory experiments suggest that the effects of ITCs have a more complex background besides generating oxidative stress, or the caused oxidative stress did not result in accumulation of hydrogen peroxide. Low persistency of ITCs also supports growth recovery similarly to the observations about fungi [49]. However, on the basis of the results, there is no doubt about the broad algicide spectrum of horseradish essential oil.

Results of microcosm experiments with natural phytoplankton assemblages show that horseradish essential oil from 7.1 × 10^−6^ % (v/v) is applicable to push back phytoplankton proliferation even in natural assemblages. The relatively weaker effect on microcosm could be explained by the organic matter content of the natural system: it was observed that the presence of organic compounds could have a serious negative impact on the antimicrobial activity of ITCs [49]. Relative abundance changes of the main taxonomical groups were chosen to assess compositional changes in microcosms. Relative abundances reflect the changes of individual numbers directly; moreover, these data hold extra information about the changes of the whole algal assemblages. They have ecological information besides the “technical” data of individual number changes. These data show not just the decreases or increases of a given group, but they show proportional changes of the assemblages in a more direct way than individual numbers. The most striking result of the microcosm experiment was the fall of the relative abundance of one group of cyanobacteria, Cyano 1, with species belonging to the order Synechococcales (Figure 3a). So, despite the result that *Synechococcus elongatus* itself was the least sensitive among the tested laboratory strains, it could be pushed out from an assemblage. Since the environmental circumstances were favorable for cyanobacterial growth, initial dominance relations (green algal dominance) and interspecific interactions could be the background of the results contrary to observations in the laboratory. This result suggests that the composition of the assemblages has a strong influence on the effects of a toxic substance, as it was observed in the case of fungal assemblages [49] and in our former microcosm studies [57]. However, this result also highlights that there is the possibility to achieve satisfactory results with the application of the horseradish essential oil, even in the case of species that showed tolerance in the laboratory. The microcosm systems (and the pond itself) can be considered as eutrophic or at least meso-eutrophic ones based on their chlorophyll-a contents [58,59]. This state did not change during the treatments, so the applied concentrations of horseradish oil had longer-term effects than aluminum or iron applied for phosphorous removal or calcium and magnesium also for phosphorous removal or as carbonates for precipitation [2,60]. On the other hand, the disadvantages of the mentioned methods (toxicity, pH influence, increase in water hardness, or production of undesirable sludge [17]) cannot be expected with using horseradish oil. However, the specification of possible side effects undoubtedly requires further investigations.

In contrast to microcosm experiments (phytoplankton assemblages), green algae showed low resistance against the treatments in the biofilm experiments. Indeed, cyanobacteria and diatoms are among early colonizers, which play a significant role in biofilm development [35]. Certain diatoms are able to settle even on the most fouling-resistant surfaces [61], so it is not a surprise that the dominant, but rather planktic green algal species (*T. obliquus*) was pushed back already at the lowest applied horseradish essential oil concentration (1.8 × 10^−6^ %). The individual number dropped down to one-fifth of the original individual number in the biofilm, so the 7.1 × 10^−6^% and higher concentration can be considered as a successful treatment against biofouling. As the results show, the remaining individuals were cyanobacteria and diatoms. The viability of the latter group is questionable because of the lack of associated xanthophylls, which are essential to the healthy physiology of these microalgae [62]. To compare the effectiveness of the applied horseradish essential oil with other compounds derived from natural sources would be too early at the current state of research. There are several compounds isolated from macroalgae (e.g., cystochloroketals [63]; chromanols [64]; alcaloids; phenolic acids [65]; dopamine [66]), but also many different extracts [67,68,69,70,71] with proved anti-algal and anti-cyanobacterial activities. There are also many types of extracts from microalgae with similar activities [72,73,74,75,76,77]. The huge advantage of plant-derived essential oils - including horseradish essential oil – is that they are renewable in nature, relatively inexpensive, available in commercial quantities (because of the food or medicine industrial background), and could have minimal toxicity (among others because of their low persistency [49]) compared to many conventionally applied chemicals [36].

## 4. Materials and Methods

### 4.1. Strains, Culturing Conditions, and Laboratory Experimental Setup

The cyanobacteria and eukaryotic algae strains used during the work, the culturing media used for their maintenance and for the experiments, and the experimental conditions are summarized in Table 2. During the experiments, the preferences of the used cyanobacterial and eukaryotic algal strains were taken into consideration. Different culturing media and temperatures were used because of the different optima of cyanobacteria and eukaryotic algae (most relevant with *C. ovata*).

For the determination of isothiocyanates in the applied horseradish essential oil, the GC-MS method described in the work of [78] was used. A 1 uL aliquot of pure horseradish essential oil, 100-fold diluted with acetone, was injected and analyzed at 1:100 split. Two main components covered the ITC content of the horseradish essential oil used for the study (87.7% ± 1.34% AITC and 2.68% ± 1.34% PEITC; Figure S2). Ethanol was used for the dilution of the essential oil. The exact amounts of the two main components in the different treatments are shown in Table 3. To check the possible effects of the diluent, cultures containing only ethanol were also applied. Control cultures were prepared without ethanol and/or essential oil. Treated cultures were supplemented with essential oil diluted in ethanol or with pure essential oil at the inoculation to reach 1.8, 3.6, 7.1, 14.3, and 28.6 × 10^−6^% (v/v) essential oil concentrations. The exposure time was 7 days for cyanobacteria and *D. salina* and 14 days for other eukaryotic algae. The growth of the cultures was monitored by measuring the chlorophyll content. Samples of 200 µL were collected daily for the measurements. Chlorophyll content was calculated on the basis of absorbance measured in 80% acetone at 663 nm [79]. Spectrophotometric measurements were performed with a Hach Lange DR 6000 UV/VIS spectrophotometer (Hach Lange GmbH, Düsseldorf, Germany).

To provide the essential oil concentrations causing 50% growth inhibition (EC_50_ values), the extent of growth inhibition (in percentage compared to control) was plotted as a function of essential oil concentrations. Trend lines were fitted to the obtained curves (second-order relationship), and from the equations of the trend lines (quadratic equations), the concentrations causing 50% inhibition were calculated.

### 4.2. Microcosm Experiments with Natural Phytoplankton Assemblages

Microcosm experiments were established in summer at the Garden Pond in the Zoo of Debrecen, which is a shallow artificial ornamental pond (average depth: 0.35 m, area: ~50 m^2^), with a constant water level maintained by the introduction of tap water. There is potted vegetation on the shore and in the pond. The microcosm system developed by our workgroup [63,84] was applied in triplicates with minor modifications (Figure S3): water samples from the pond were filled into borosilicate glass beakers (2 l in each one). One beaker served as control (without the addition of ethanol or essential oil), one contained only ethanol in 28.6 × 10^−6^ % (v/v), and the others were used to test five experimental treatments, containing 1.8, 3.6, 7.1, 14.3, and 28.6 × 10^−6^ % (v/v) essential oil. The beakers were placed in plastic baskets, and the baskets were placed into the pond 20 cm deep. The baskets had thin translucent (polyester) tops to allow photosynthesis and gas exchange but prevent the beakers from damage (the distance between the water surface of the beakers and the translucent top was at least 8 cm). There was no exchange between the beakers’ contents and the pond water, but the baskets containing the beakers allowed the pond water to flow in and surround the beakers, resulting in efficient heat exchange with the pond. Environmental parameters (water temperature, pH, conductivity, O_2_ concentration, and saturation) were measured by an HQ30d multimeter (Hach Lange GmbH, Düsseldorf, Germany) every 48 h during 14 days. Samples of 20 mL were collected at the same times from all microcosms for counting algal individuals, taxonomical identification, and chlorophyll-a measurements. The water level in the microcosms was maintained by adding tap water before sampling.

Aliquots of 5 mL were used for chlorophyll-a content measurements. Samples were centrifuged (6000× g, 10 min, Beckman Avanti J-25, Beckman Industries Inc., Fullerton, California, USA), the supernatants were removed, pellets were lyophilized (Christ Alpha 1e2 LD plusfreeze-dryer equipment, Martin Christ Gefriertrocknungsanlagen GmbH, Osterode am Harz, Germany) and their weights were measured (Ohaus Adventurer™ Pro analytical scale, Ohaus Corporation, Parsippany, New Jersey, USA). The chlorophyll-a content of the weighted pellets was measured spectrophotometrically using the hot methanolic extraction method [64]. To provide the essential oil concentrations causing 50% growth inhibition (EC_50_ values), the same method was applied as in the case of laboratory experiments. Aliquots of 15 mL of the collected samples were stored in Lugol’s solution for taxa identification and counting the number of algal individuals. The samples were processed according to the European Standard EN 15204:2006 [85]. An Olympus CKX31 reversed microscope and 400× magnification was used for counting and taxa identification. The limit of detection was 0.7×10^6^ individuals per liter. The identified taxa in natural assemblages (microcosms) were taxonomically grouped using AlgaeBase [86], and relative abundances were calculated. Relative taxa were grouped together, all in all, the following eight groups were created (each with higher relative abundances than 3% at some time of the exposition): Cyano 1 and 2 (Synechococcales and Chroococcales, respectively); Diatom 1 and 2 (Bacillariophyceae and Mediophyceae, respectively) and Green 1–4 (Trebouxiophyceae, Chlorophyceae, Conjugatophyceae, and Carophyceae + Klebsormidiophyceae, respectively).

### 4.3. Algal Biofilm Experiments with Natural Benthic Algal Assemblages

In algal biofilm experiments, a sample from natural biofilm formed in an aquaponic culture was used to produce the algae “coatings”. The inoculum constituted ~45% of diatoms, ~45% unicellular coccoid cyanobacteria, and ~10% coccoid green algae. Pieces of 5×15 cm white tiles were used as model surfaces. The tiles were placed in glass tubs filled with Jaworski’s Medium [82] and kept at room temperature (~23 °C) under natural irradiation. The tubs were covered with transparent plastic (polyester) lids to minimize evaporation. After the development of biofilms (after 4 weeks), the tiles were placed into glass tubs filled with Jaworski’s Medium [82] without essential oil (control) and with 1.8, 7.1, and 28.6 × 10^−6^ % (v/v) essential oil dissolved in ethanol. The time of the exposition was 6 weeks. At the end of the exposure time, the biofilms were removed from the entire surfaces (75 cm^2^ per tile) with a fine-edged spatula, resuspended in 20 mL of culturing medium, and preserved with Lugol’s solution. Algal individuals were counted and identified from the preserved samples by the same method as it is described in the case of microcosm experiments. Knowing the individual numbers, it was possible to characterize the quantitative relations of the biofilms to a given surface. To provide the essential oil concentrations causing 50% growth inhibition (EC_50_ values), the same method was applied as in the case of laboratory and microcosm experiments.

### 4.4. Data Analyses

All experiments were performed in triplicates (as single experiments with 3 repetitions of all individual groups). Analysis of covariance of means of chlorophyll content data (one-way ANCOVA, Past 2.17b, [87]) was used to show differences among the growth trends of control and treated cultures or assemblages.

To present the compositional changes, individual number data of the groups were averaged within the three replicates, two-way analysis of variance (two-way ANOVA, Past 2.17b [87]) with Tukey post-hoc test was applied to evaluate the differences among relative abundances in time and of the certain composition of control and treated assemblages.

Analysis of variance (one-way ANOVA, Past 2.17b [87]) with Tukey post-hoc test was applied to evaluate the differences among individual numbers and composition of control and treated biofilms.

## 5. Conclusions

Increased proliferation of algae: blooms of planktic species or biofouling by benthic ones is a current problem in natural as well as in artificial water bodies. Effective handling of these phenomena becomes more and more urgent. Chemicals derived from natural sources, which could be effective in low concentrations and are biodegradable, may have an advantage over conventional chemical treatments [17]. In this study, the anti-algal effects of AITC- and PEITC-containing horseradish essential oil were tested in laboratory experiments, on natural phytoplankton assemblages, and on semi-artificial biofilm. The results show that acute treatment can significantly reduce the viability of laboratory strains of potentially bloom-forming cyanobacteria and eukaryotic algae. Results of microcosm experiments with natural phytoplankton assemblages show that horseradish essential oil from 7.1 × 10^−6^ % (v/v) is applicable to push back phytoplankton proliferation even in natural assemblages. The regeneration ability of the assemblages suggests the necessity of weekly treatment for successful control of phytoplankton proliferation. In the biofilm, the individual number was dropped down to one-fifth of the original individual number, so 7.1 × 10^−6^ % (v/v) and higher concentration of the essential oil can be considered as successful treatment also against biofouling. Horseradish is well known, widely used with a well-established industrial background [40,88]. The essential oil used in this study can be produced even from processed horseradish vegetable waste [89], so it can be a promising basis for the development of anti-algal products from natural sources.

## Figures and Tables

**Table 1:** Horseradish essential oil concentrations (× 10^−6^ v/v %) causing 50% growth inhibition on the 4^th^, 7^th^, and 14^th^ day of the exposition based on chlorophyll-a content of the cultures. Mean values and standard deviations are indicated (n = 3).

Strains	EC_50_ (× 10^−6^ v/v % Horseradish Essential Oil)		
	4^th^ Day	7^th^ Day	14^th^ Day
*Synechococcus elongatus*	7.66 ± 2.26	7.95 ± 0.48	-
*Microcystis aeruginosa*	4.97 ± 0.25	5.07 ± 0.38	-
*Cylindrospermopsis raciborskii*	4.77 ± 2.87	4.13 ± 3.07	-
*Dunaliella salina*	1.33 ± 0.07	1.47 ± 0.08	-
*Cryptomonas ovata*	1.81 ± 0.41	1.63 ± 0.47	2.07 ± 0.39
*Chlorella sorokiniana*	1.57 ± 0.05	1.67 ± 0.02	1.77 ± 0.05
*Chlorococcum* sp.	2.37 ± 0.49	2.69 ± 0.12	2.81 ± 0.05
*Desmodesmus communis*	5.67 ± 0.24	4.17 ± 1.21	4.76 ± 0.27

**Table 2:** Culturing conditions of cyanobacteria and eukaryotic algae isolates used to study the algaecide effect of horseradish essential oil.

Prokaryotes	Culturing Medium	Culture Parameters
*Synechococcus elongatus*	Allen Medium [80]	100 mL Erlenmeyer flask on shaker (90 rpm), 35 mL final volume, 28 °C, continuous irradiation (80 µmol photons m^−2^ s^−1^).
*Microcystis aeruginosa*		
*Cylindrospermopsis raciborskii*	Allen Medium (without nitrate)	
Eukaryotes		
*Dunaliella salina*	Johnson’s Medium [81]	100 mL Erlenmeyer flask on shaker (90 rpm), 35 mL final volume, 28 °C, continuous irradiation (80 µmol photons m^−2^ s^−1^).
*Cryptomonas ovata*	Jaworski’s Medium [82]	100 mL Erlenmeyer flask on shaker (90 rpm), 50 mL final volume, 24 °C, continuous irradiation (40 µmol photons m^−2^ s^−1^).
*Chorella sorokiniana*	Bold’s Basal Medium [83]	
*Chlorococcum* sp.		
*Desmodesmus communis*		

**Table 3:** Horseradish essential oil concentrations (v/v %) in the different treatments and the allyl-isothiocyanate (AITC) and phenethyl-isothiocyanate (PEITC) contents (µM).

Treatments (v/v % Horseradish Oil)	µM AITC	µM PEITC
1.8 × 10^−6^	0.78 ± 0.012	0.016 ± 0.008
3.6 × 10^−6^	1.57 ± 0.025	0.032 ± 0.016
7.1 × 10^−6^	3.13 ± 0.05	0.064 ± 0.032
14.3 × 10^−6^	6.25 ± 0.10	0.128 ± 0.064
28.6 × 10^−6^	12.51 ± 0.20	0.257 ± 0.128

## Data Availability

The data presented in this study are available on request from the corresponding author.

## Supplementary Materials

The following are available online at https://www.mdpi.com/article/10.3390/plants10081550/s1, Table S1: The most commonly observed genera or species in the microcosms (with higher relative abundances than 3% of the corresponding taxa at some time of the exposition. Figure S1: Development of biofilms in (a) control and in (b) 1.8 × 10^−6^; (c) 7.1 × 10^−6^; and (d) 28.6 × 10^−6^ % (v/v) horseradish essential oil treatment during six weeks exposition, Figure S2: Chromatogram of the horseradish essential oil used in the study. AITC: Allyl-isothiocyanate, PEITC: phenethyl-isothiocyanate, Figure S3: Sketch of the experimental setup in microcosm experiments.
