# Supplementary material for: Horseradish Essential Oil as a Promising Anti-Algal Product for Prevention of Phytoplankton Proliferation and Biofouling
Source: Plants (Basel). 2021 Jul 28;10(8):1550. doi: 10.3390/plants10081550 (PMC8400301; doi:10.3390/plants10081550)
Supplement: Supplementary file 1 [file plants-10-01550-s001.zip › plants-1318339-supplementary.pdf]

Plants

Horseradish essential oil as a promising anti-algal product for prevention of phytoplankton proliferation and biofouling

István Bácsi [1,*], Sándor Gonda [2], Zsuzsanna Nemes-Kókai [3,4,5], Viktória B-Béres [3], and Gábor Vasas [2]

[1] University of Debrecen, Department of Hydrobiology, H-4032 Debrecen, Egyetem tér 1, Hungary; istvan.bacsi@gmail.com
[2] University of Debrecen, Department of Botany, H-4032 Debrecen, Egyetem tér 1, Hungary; gondasandor@gmail.com
[3] Centre for Ecological Research, Institute of Aquatic Ecology, Department of Tisza Research, H-4026 Debrecen, Bem tér 1, Hungary; kokai.zsuzsanna@ecolres.hu; beres.viktoria@gmail.com
[4] University of Debrecen, Department of Ecology, H-4032 Debrecen, Egyetem tér 1, Hungary; kokai.zsuzsanna@ecolres.hu
[5] Pál Juhász-Nagy Doctoral School of Biology and Environmental Sciences, University of Debrecen, Egyetem sqr. 1., H-4032 Debrecen, Hungary; kokai.zsuzsanna@ecolres.hu
* Correspondence: istvan.bacsi@gmail.com; Tel.: +36 52 512 900 / 22634

Table S1. The most commonly observed genera or species in the microcosms (with higher relative abundances than 3% of the corresponding taxa at some time of the exposition).

**Group**	**Taxonomic group**	**Genera or species**
Cyano 1	Synechococcales	*Aphanocapsa* sp.<br>*Merismopedia* sp.<br>*Planktolyngbya* sp.<br>*Synechococcus* sp.
Cyano 2	Chroococcales	*Aphanothece* sp.<br>*Chroococcus* sp.<br>*Gloeothece* sp.<br>*Microcystis* sp.
Diatom 1	Bacillariophyceae	*Fragilaria capucina*<br>*Navicula* sp.<br>*Nitzschia* sp.
Diatom 2	Mediophyceae	*Cyclotella* sp.
Green 1	Trebouxiophyceae	*Chlorella* sp.<br>*Crucigeniella apiculata*<br>*Koliella* sp.<br>*Oocystis lacustris*
Green 2	Chlorophyceae	*Coelastrum morus*<br>*Coelastrum reticulatum*<br>*Desmodesmus communis*<br>*Desmodesmus spinosus*<br>*Golenkinia radiata*<br>*Kirchneriella irregularis*<br>*Kirchneriella obesa*<br>*Monoraphidium pusillum*<br>*Tetraedron caudatum*
Green 3	Conjugatophyceae	*Cosmarium* sp.<br>*Staurastrum* sp.<br>Unidentified filamentous species
Green 4	Carophyceae<br>Klebsormidiophyceae	*Closterium* sp.<br>*Elakatothrix genevensis*

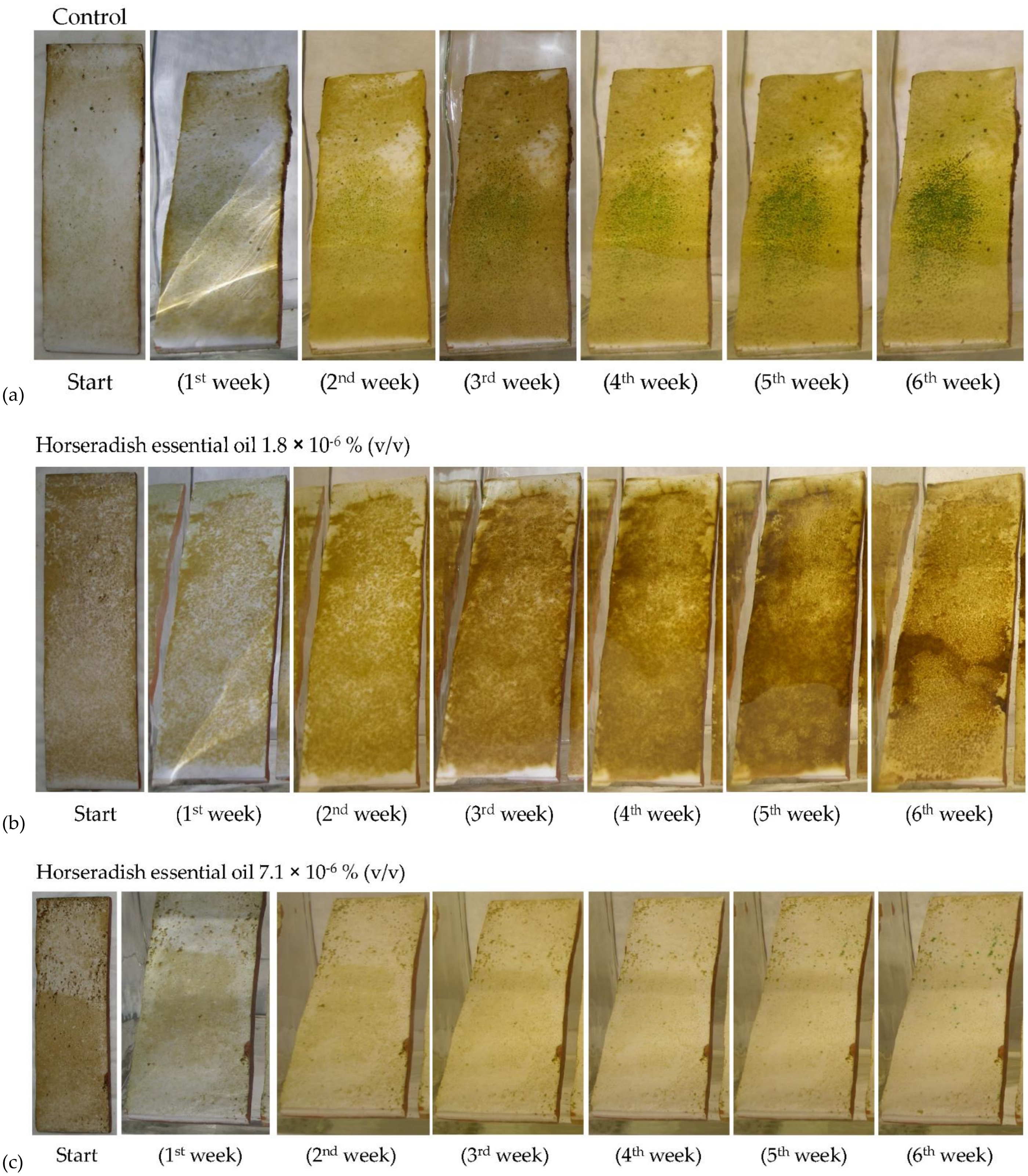
Control
Start
(1st week)
(2nd week)
(3rd week)
(4th week)
(5th week)
(6th week)
(a)
Horseradish essential oil 1.8 × 10-6 % (v/v)
Start
(1st week)
(2nd week)
(3rd week)
(4th week)
(5th week)
(6th week)
(b)
Horseradish essential oil 7.1 × 10-6 % (v/v)
Start
(1st week)
(2nd week)
(3rd week)
(4th week)
(5th week)
(6th week)
(c)

Horseradish essential oil 28.6 × 10^{-6} % v/v

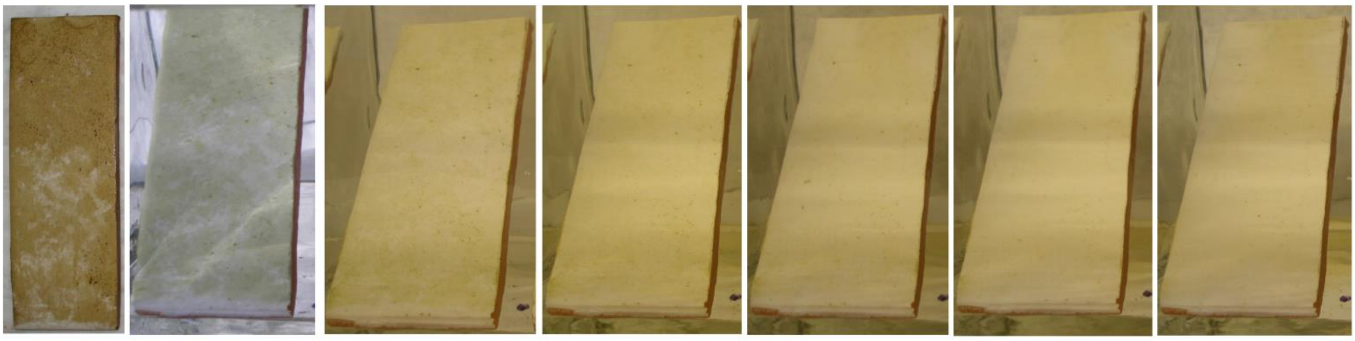

(d) Start (1st week) (2nd week) (3rd week) (4th week) (5th week) (6th week)

Figure S1. Development of biofilms in (a) control and in (b) 1.8 × 10^{-6}; (c) 7.1 × 10^{-6} and (d) 28.6 × 10^{-6} % (v/v) horseradish essential oil treatment during six weeks exposition.

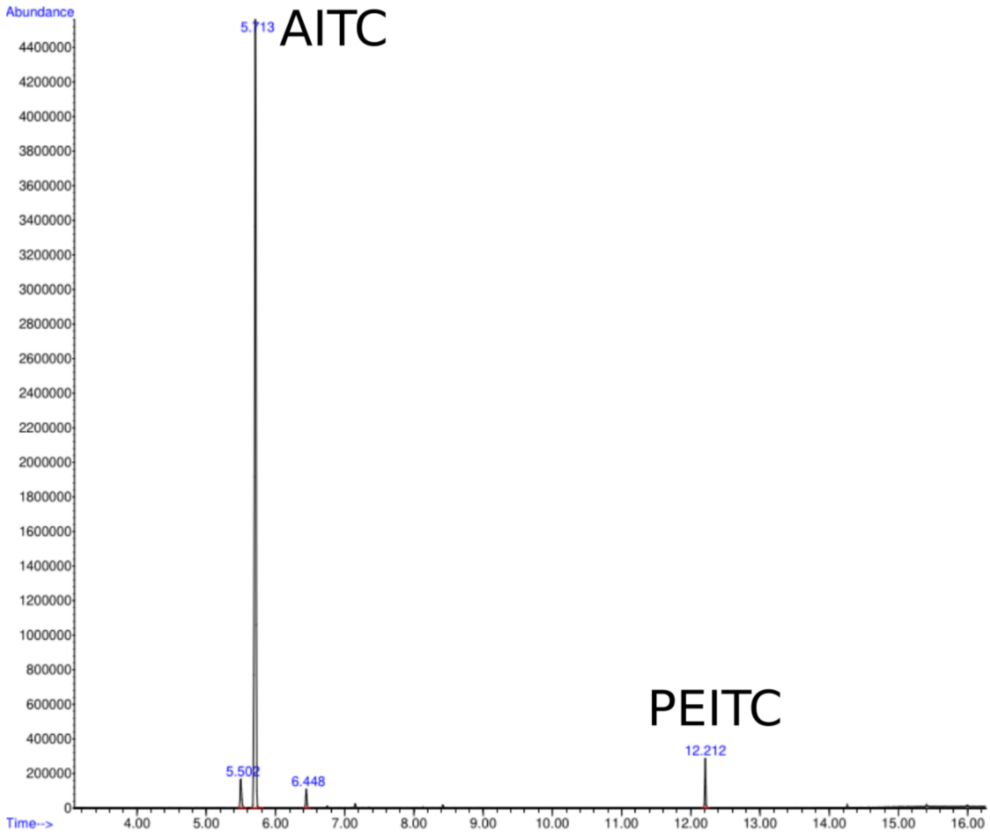

Figure S2. Chromatogram of the horseradish essential oil used in the study. AITC: Allyl-isothiocyanate, PEITC: phenethyl-isothiocyanate.

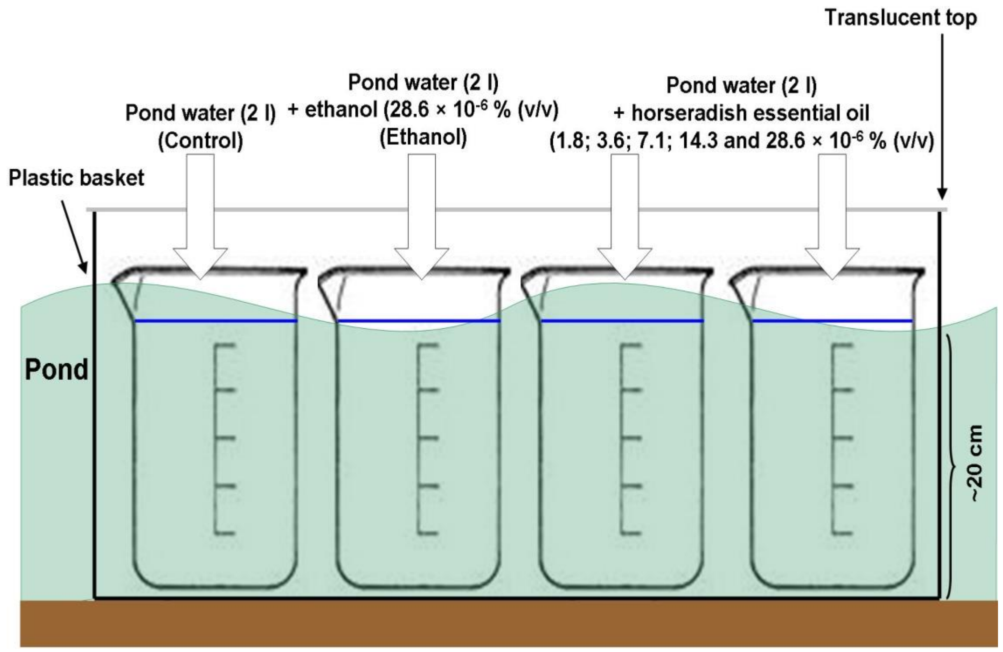

Figure S3. Sketch of the experimental setup in microcosm experiments.
